# Genomic Survey of Carbon Monoxide Dehydrogenases Reveals Their Widespread Distribution in Marine Habitats

**DOI:** 10.1111/1758-2229.70375

**Published:** 2026-06-03

**Authors:** Nipa Chongdar, Anand Goyal, Samir R. Damare

**Affiliations:** ^1^ School of Interdisciplinary Life Sciences Indian Institute of Technology Goa Ponda India; ^2^ CSIR‐National Institute of Oceanography Dona Paula India; ^3^ National Institute of Technology, Warangal Hanumkonda India

**Keywords:** carbon monoxide oxidation, carboxydotroph, database analysis, marine microbes, metalloproteins

## Abstract

Most carbon monoxide (CO) produced in the ocean is consumed by microorganisms encoding carbon monoxide dehydrogenases (CODHs), thereby significantly reducing the flux of CO from the ocean to the atmosphere. CODHs are of two types based on the metal content of their active sites: the oxygen‐sensitive, nickel‐containing Ni‐CODH and the oxygen‐tolerant, molybdenum–copper‐containing Mo‐CODH. Although CODHs have been reported from specific marine environments, their combined distribution across ocean ecosystems remains unclear. Here, we analyzed the NCBI non‐redundant protein database and identified 1969 Ni‐CODH and 864 Mo‐CODH genes from marine prokaryotes spanning diverse oceanic ecosystems. Using metagenomic analyses across three marine biomes, we showed that oxygen availability selectively constrains Ni‐CODH gene abundance, but not Mo‐CODHs. Thus, Ni‐CODHs are restricted to oxygen‐limited niches, while Mo‐CODHs occur across both oxygenated and oxygen‐limited marine environments. Phylogenetic analyses indicated that all previously described CODH clades are represented in the marine ecosphere, highlighting their evolutionary diversity. Genome context analyses suggest that approximately 50% of the marine Ni‐CODH potentially participate in carbon fixation via the Wood‐Ljungdahl pathway, whereas most marine Mo‐CODH likely contribute to the supplementary energy conservation. Together, these results provide an integrated view of CODH distribution and potential function in marine ecosystems.

## Graphical abstract

Protein sequence surveys show that nickel‐ and molybdenum/copper‐containing carbon monoxide dehydrogenases (CODHs) are widespread in the ocean. Phylogenetic and genome context analyses reveal that marine CODHs are highly diverse in both evolutionary history and functional organisation.

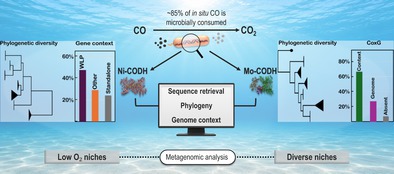

## 1 Introduction

**FIGURE 1:**
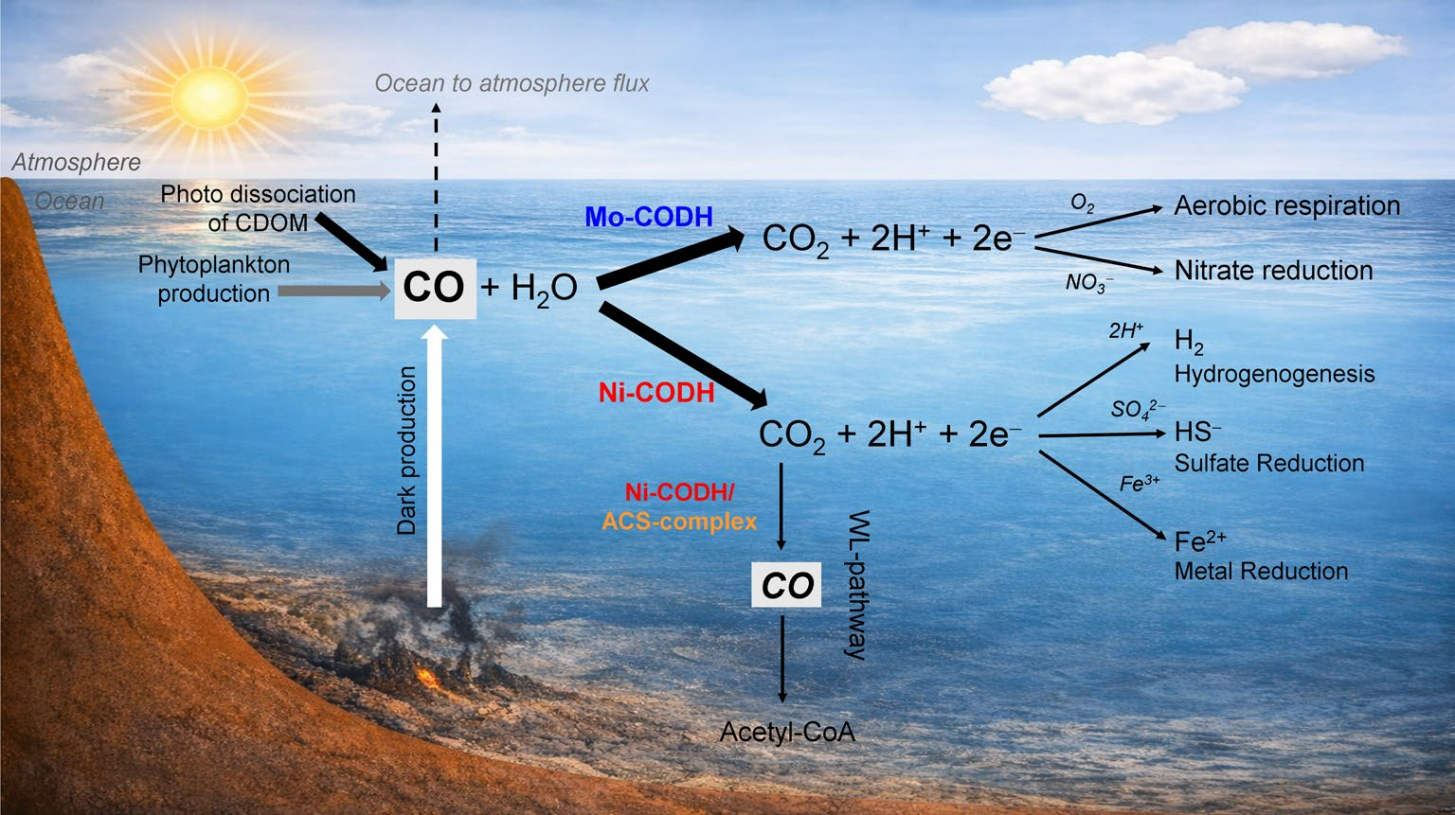
Overview of carbon monoxide (CO) production and microbial consumption in the marine environment. CO is generated in seawater through photochemical and dark processes and is efficiently consumed by microorganisms. Microbial CO consumption is catalysed by CODHs. Electrons released during CO oxidation can be transferred to terminal electron acceptors, such as protons, sulphate, nitrate, metals or oxygen, supporting energy conservation in both anaerobic and aerobic carboxydotrophs. Bidirectional Ni‐CODHs, often associated with the acetyl‐CoA synthase (ACS) complex, can catalyse the reduction of CO_2_ to CO, a key intermediate of the Wood–Ljungdahl (WL) pathway for acetyl‐CoA formation.

The ocean is a natural source of carbon monoxide (CO), a trace gas that modulates tropospheric chemistry by depleting hydroxyl radicals, thereby indirectly influencing methane lifetime and promoting ozone formation (Swinnerton et al. 1970; Conte et al. 2019; Lelieveld et al. 2016). In oceans, CO is produced primarily through the photochemical degradation of chromophoric dissolved organic matter (CDOM) in the sunlit euphotic zone (Zuo and Jones 1995; Stubbins et al. 2006; Xie and Zafiriou 2009) (Figure 1). In addition, non‐photochemical or ‘dark’ CO production occurs throughout the water column via microbial metabolism, enzymatic breakdown of pigments and abiotic thermal or oxidative transformation of dissolved organic matter, suggesting that these processes may contribute to CO availability below the photic zone (Kettle 2005; Xie et al. 2005; Zhang et al. 2008). Beyond the water column, ocean sediments were also identified as potential sources of CO, due to microbial activities and degradation of organic matter (Qi et al. 2025; Kleiner et al. 2015).

Despite CO being sparingly soluble in water (~27 mg/L at 25°C), only a minor fraction of the CO produced in the ocean is released into the atmosphere. Instead, marine microorganisms rapidly consume 65%–96% of in situ CO, making microbial oxidation the most dominant CO sink process in the marine environment (Conrad et al. 1982; Conte et al. 2019; Xu et al. 2023; Yang et al. 2024). The central role of microbial CO consumption has motivated the investigation of CO‐oxidising prokaryotes in the ocean.

CO‐oxidising prokaryotes are of two types: carboxydotrophs, which grow using CO as the sole source of carbon and energy, and carboxydovores, which oxidise CO to supplement energy metabolism (King and Weber 2007). These microorganisms catalyse the conversion of CO to CO_2_ using specialised metalloenzymes called carbon monoxide dehydrogenases (CODHs) (Figure 1) (Jeoung et al. 2019; Bährle et al. 2023). Two structurally and phylogenetically distinct types of CODHs are known. Nickel‐containing CODHs (Ni‐CODH) are the O_2_‐sensitive, homodimeric enzymes containing a nickel (Ni)–iron (Fe)–sulphur (S) cubane at the active centre, whereas Mo‐CODHs are O_2_‐tolerant, heterotrimeric enzymes with molybdenum (Mo) and copper (Cu) at the active centre of the catalytic subunit (Figure S1A) (King and Weber 2007; Can et al. 2014; Jeoung et al. 2019; Bährle et al. 2023). Functionally, Mo‐CODHs catalyse only CO oxidation, whereas some Ni‐CODHs, particularly those associated with the acetyl‐CoA synthase (ACS) complex, can also reduce CO_2_ to CO (Can et al. 2014; Adam et al. 2018). Given the highly negative redox potential of the CO_2_/CO couple (−520 mV vs. the standard hydrogen electrode at pH 7 and 25°C), CO serves as an excellent source of electrons (Appel et al. 2013). Consequently, the electrons generated by CODH‐mediated CO oxidation are utilised in cellular energy conservation and carbon metabolism (Figure 1).

In organisms encoding Ni‐CODH, electrons produced by CO oxidation can be used to reduce a range of terminal electron acceptors, such as protons (H^+^), sulphate (SO_4_^2−^) or ferric‐iron (Fe^3+^), to facilitate ATP generation (Figure 1) (Fukuyama et al. 2020; Diender et al. 2015; Zavarzina et al. 2007; Henstra et al. 2007). In many anaerobes, where Ni‐CODHs function as part of the ACS complex, they reduce CO_2_ to CO, an essential intermediate for the Wood–Ljungdahl (WL; reductive acetyl‐CoA) pathway, to generate acetyl‐CoA (Can et al. 2014). In contrast, Mo‐CODH–encoding microbes transfer electrons from CO oxidation into the quinone pool (Wilcoxen et al. 2011). Under oxygenated conditions, these electrons are accepted by oxygen, whereas under oxygen‐limited conditions, they can be redirected to nitrate (Cordero et al. 2019; Imaura et al. 2023; G. M. King 2006). Therefore, such diverse electron‐transfer strategies connect CO oxidation to carbon, hydrogen, sulphur and nitrogen cycling processes in marine ecosystems.

In 1982, Conrad and Seiler first demonstrated that seawater bacteria actively consume CO (Conrad et al. 1982). Subsequent culture‐based studies expanded this finding by identifying Mo‐CODH‐containing bacteria, predominantly in coastal and surface waters (Moran et al. 2004, 2007; Tolli et al. 2006; Cunliffe 2011). Metagenomics and metaproteomics surveys identified Mo‐CODH genes across more diverse marine habitats, including open‐ocean water columns, seagrass and deep‐sea sediments (Smedile et al. 2013; Acinas et al. 2021; Martin‐Cuadrado et al. 2009; Quaiser et al. 2011; Lappan et al. 2023; Kleiner et al. 2015; Saunders et al. 2022; Martín‐Cuadrado et al. 2007). Several Ni‐CODH‐expressing species were identified in hydrothermal or subseafloor environments through culture‐based and metagenomic studies (Yoneda et al. 2013; Sokolova et al. 2001, 2004; Rother and Metcalf 2004; Beeder et al. 1994; Kozhevnikova et al. 2016; Slobodkin et al. 2019; Hoshino and Inagaki 2017; Carr et al. 2019; Baker et al. 2016).

Despite these advances, a unified assessment of the diversity, distribution and ecological roles of both Ni‐ and Mo‐CODHs across the global ocean remains lacking. A recent global database survey on the biome‐specific distribution of CODHs focused exclusively on Ni‐CODHs and grouped the marine‐derived sequences within a broad aquatic habitats category (Inoue et al. 2022). Here, we conducted an extensive analysis of CODH genes within the marine realm using a protein–sequence–based data‐mining approach. By screening publicly available protein sequences derived from metagenome‐assembled genomes (MAGs) and isolated genomes, we constructed an ocean‐wide inventory of CODHs. Our analyses provide new insights into the ecological distribution, phylogenetic diversity and potential functional roles of CODHs in marine CO‐oxidising prokaryotes.

## 2 Materials and Methods

### 2.1 CODH Sequence Retrieval

Ni‐CODH and Mo‐CODH (large subunit) protein sequences were obtained from the National Centre for Biotechnology Information (NCBI) non‐redundant database (as of July 2023) using a BLASTp search (Altschul et al. 1990). Ni‐CODHs are of two types: the CooS type, which is predominantly found in bacteria, and the CdhA‐type, which is mostly found in archaea. Although the residues ligating Ni, Fe active centres are primarily conserved in CooS‐ and CdhA‐type Ni‐CODHs, the overall sequence identity of CdhA‐type and CooS‐type CODHs is low, and CdhA‐type Ni‐CODHs harbour two extra iron–sulphur clusters compared to the CooS‐type (Techtmann et al. 2012). To ensure comprehensive retrieval, BLASTp searches were performed using representative Ni‐CODH sequences spanning all previously described clades (Inoue et al. 2019), encompassing both CooS‐ and CdhA‐type enzymes and including sequences with divergent active‐site features (WP_011343033.1, WP_011342982.1, WP_026514536.1, WP_039226206.1, WP_011305243.1, WP_012571978.1, WP_007288589.1 and OGP75751.1). To retrieve the Mo‐CODH sequences from the databases, Mo‐CODH large subunit (catalytic subunit, CoxL) sequences were used as queries (WP_013913730.1, WP_003892166.1 and WP_00606799.1) (Figure S2). For both datasets, sequences with low query coverage (amino acid length < 400 for Ni‐CODH and < 700 for Mo‐CODH) and low bit scores (< 200) were excluded. Redundant (100% identical) Ni‐CODH and Mo‐CODH (CoxL) sequences were removed using Jalview (Waterhouse et al. 2009) before further curation (Figure S2).

Finally, the retrieved sequences were validated based on conserved active‐site features required to coordinate the active‐site cofactors. Ni‐CODH candidates were curated to retain only sequences containing all five C‐cluster–coordinating cysteine residues, as well as no deletion of B‐and D‐clusters. Mo‐CODH sequences were validated based on the presence of the conserved active‐site motif (typically reported as AYXCSFR), allowing for limited variation (e.g., substitutions at the first and sixth positions).

### 2.2 Isolation Metadata Retrieval

Isolation source or habitat information for genomes encoding Ni‐CODH and Mo‐CODH sequences was obtained using the BioSample identifier linked to each protein entry (feature table, ‘BioSample’ field in GenBank format). The BioSample database provides structured metadata fields, including ‘isolation source’ and ‘geographic location’, which were used to classify sequences as marine and to assign them to specific marine biomes (e.g., hydrothermal vent, marine sediment). BioSample identifiers were not available for proteins with RefSeq identifiers. For these entries, habitat information was retrieved from corresponding publication records of the source organisms. Ambiguous or non‐specific entries were excluded from downstream analysis.

### 2.3 Metagenomic Short Read Analysis

To perform metagenomic short read analysis, raw metagenomic sequencing data were retrieved from the NCBI SRA database (BioProjects: PRJNA247822, PRJNA1115057, PRJNA707313 and PRJNA1054206) (Leinonen et al. 2011). The downloaded SRA format sequence data were converted to paired‐end FASTQ files using fasterq, followed by adapter trimming and quality filtering using fastp (Chen et al. 2018). For downstream analysis, only the quality‐filtered paired‐end reads were retained. Metabolic annotation of these high‐quality reads was performed using DIAMOND v2.1.13 (Buchfink et al. 2021) by aligning reads against a custom database comprising sequences of Ni‐ and Mo‐CODHs. For each read, only the best‐scoring hit was retained by restricting searches to a single target sequence and a single high‐scoring segment pair (max‐target‐seqs 1, max‐hsps 1) (Buchfink et al. 2021). A minimum sequence identity threshold of 50% was applied to identify high‐confidence matches.

The DIAMOND hits were further filtered by retaining only those alignments with a minimum amino‐acid alignment length of 32 residues. Further gene‐specific percent identity filters, at 60% for Mo‐CODH (*coxL*) and 50% for Ni‐CODH (*cooS* and *cdhA*), were applied to improve specificity. The alignments passing through all filtering steps were counted and normalised for gene length and read depth to calculate RPKM (Reads Per Kilobase per Million reads). To estimate the proportion of community members encoding CODH genes, the ‘average gene copies per organism’ was calculated by dividing CODH RPKM by the mean RPKM of 14 universal single‐copy ribosomal proteins, following the approach described by Lappan et al. (2023).

### 2.4 Phylogenetic Analysis of CODH Sequences

The taxonomic assignment of the genomes was performed using GTDB‐tk v2.5.2, with reference data version R226 (Chaumeil et al. 2022). For phylogenetic analysis, marine Ni‐CODH and Mo‐CODH protein sequences were aligned separately using the E‐INS‐I method in the MAFFT programme (Katoh 2002). The aligned sequences were curated using trimAl (automatic) from the NGphylogeny webserver to remove the poorly aligned regions from the multiple sequence alignment (Capella‐Gutiérrez et al. 2009; Lemoine et al. 2019). Trimmed alignments were used to construct phylogenetic trees using the maximum‐likelihood method in IQtree version 2.3.4, with the LG + I + R10 and LG + F + I + R9 models identified by ModelFinder for Ni‐CODH and Mo‐CODH sequences, respectively (Nguyen et al. 2015; Kalyaanamoorthy et al. 2017). The robustness of the tree topology was evaluated using Ultrafast Bootstrapping (UFBoot) in IQTree, based on 1000 replicates. The resulting phylogenetic trees were visualised and analysed using the R package *ggtree*, version 3.8.2 (Yu et al. 2017).

### 2.5 Genomic Context Analysis

The genomic contexts of Ni‐CODH and Mo‐CODH were analysed using the method described by Inoue et al. (2019) (Techtmann et al. 2012). Briefly, operons containing Ni‐ and Mo‐CODH‐encoding genes were obtained from NCBI Identical Protein Groups. The coding sequences (CDS) within 15 genes upstream and downstream of the CODH genes were annotated by the cluster of orthologous groups of proteins (COG) numbers (Tatusov et al. 2000), using reverse‐PSI (RPS)‐BLAST (*e*‐value < 10^−6^) against the NCBI conserved domain database (Lu et al. 2020). We used this information to classify marine Ni‐CODHs into different functional classes. This method was also used to determine the order of the *coxM*, *coxS* and *coxL* genes in Mo‐CODH operons and identify the nearby *coxG* genes.

To identify distantly located *acsB/cdhA* or *coxG* genes in genomes that lack them in their immediate operonic context, the complete protein datasets derived from these genomes were analysed. Protein sequences corresponding to *acsB/cdhA* (COG1614) and *coxG* (COG3427) were retrieved from the eggNOG database. They were curated by length filtering with seqkit, retaining sequences of 350–762 amino acids for COG1614 and 150–450 amino acids for COG3427. The curated sequences were aligned using MAFFT, and profile HMMs were constructed using hmmbuild (HMMER v3.3.2). These HMM profiles were used to search genome‐derived protein datasets using hmmsearch. Only hits with *E*‐values ≤ 1 × 10^−30^ were retained to identify high‐confidence homologues of *acsB/cdhA* or *coxG*. Genomes were classified as positive for *acsB/cdhA* or *coxG* if at least one such high‐confidence hit was detected.

## 3 Results

### 3.1 Construction of Marine CODH Datasets

The BLASTp search against the NCBI non‐redundant (nr) database retrieved 10,324 Ni‐CODH and 33,558 Mo‐CODH sequences. The retrieved Ni‐CODH sequences were curated by removing hits that lacked the essential amino acids required to coordinate the catalytic C‐cluster (Dobbek et al. 2001). In earlier studies, sequences with alterations in the C‐cluster coordinating cysteine at position 295 (as identified in the structure of *Carboxydothermus hydrogenoformans* Ni‐CODH II, PDB code 1su6) were also classified as Ni‐CODHs (Inoue et al. 2019). However, recent findings have shown that Ni‐CODH‐V, which contains a glutamic acid in place of cysteine at this position, coordinates an iron‐oxo hybrid cluster rather than the nickel and iron clusters characteristic of Ni‐CODHs, and does not catalyse CO oxidation or CO_2_ reduction reactions (Jeoung et al. 2022). Therefore, we excluded any retrieved sequences that showed mutations in the C‐cluster ligating cysteine residues.

Mo‐CODHs are heterotrimeric proteins of which the largest subunit, CoxL, binds the molybdopterin active site responsible for CO oxidation (Dobbek et al. 1999). We have retrieved and analysed the CoxL subunit sequences. Mo‐CODHs occur as two closely related forms, Form I and Form II, which share several conserved motifs and cluster together phylogenetically within the molybdenum hydroxylase family (King and Weber 2007). However, only Form I enzymes contain the diagnostic (A/S)YRCS(F/L)R motif within the catalytic pocket, whereas Form II features an AYRGAGR motif, a sequence also found in other molybdenum hydroxylases (King and Weber 2007). Form I Mo‐CODHs have been experimentally validated as CO‐oxidising enzymes, while the activity of Form II remains unresolved (King and Weber 2007; Cunliffe 2011; Cordero et al. 2019). Therefore, to restrict the dataset to true aerobic CO dehydrogenases, we retained only Form I Mo‐CODHs, identified by the diagnostic (A/S)YRCS(F/L)R active‐site motif (Cunliffe 2011; King and Weber 2007).

Next, environmental metadata were collected for each genome or MAGs containing the CODH genes to segregate marine‐origin CODHs. The final marine dataset comprised 1969 Ni‐CODH sequences (from 408 genomes and 1561 MAGs) and 864 Form I Mo‐CODH sequences (from 340 genomes and 524 MAGs) (Data S1 and S2). With this curated dataset, we investigated the ecological distribution and phylogenetic diversity of Ni‐ and Mo‐CODHs in the ocean ecosystems.

### 3.2 Ecological Distribution of CODH‐Coding Marine Organisms

**FIGURE 2:**
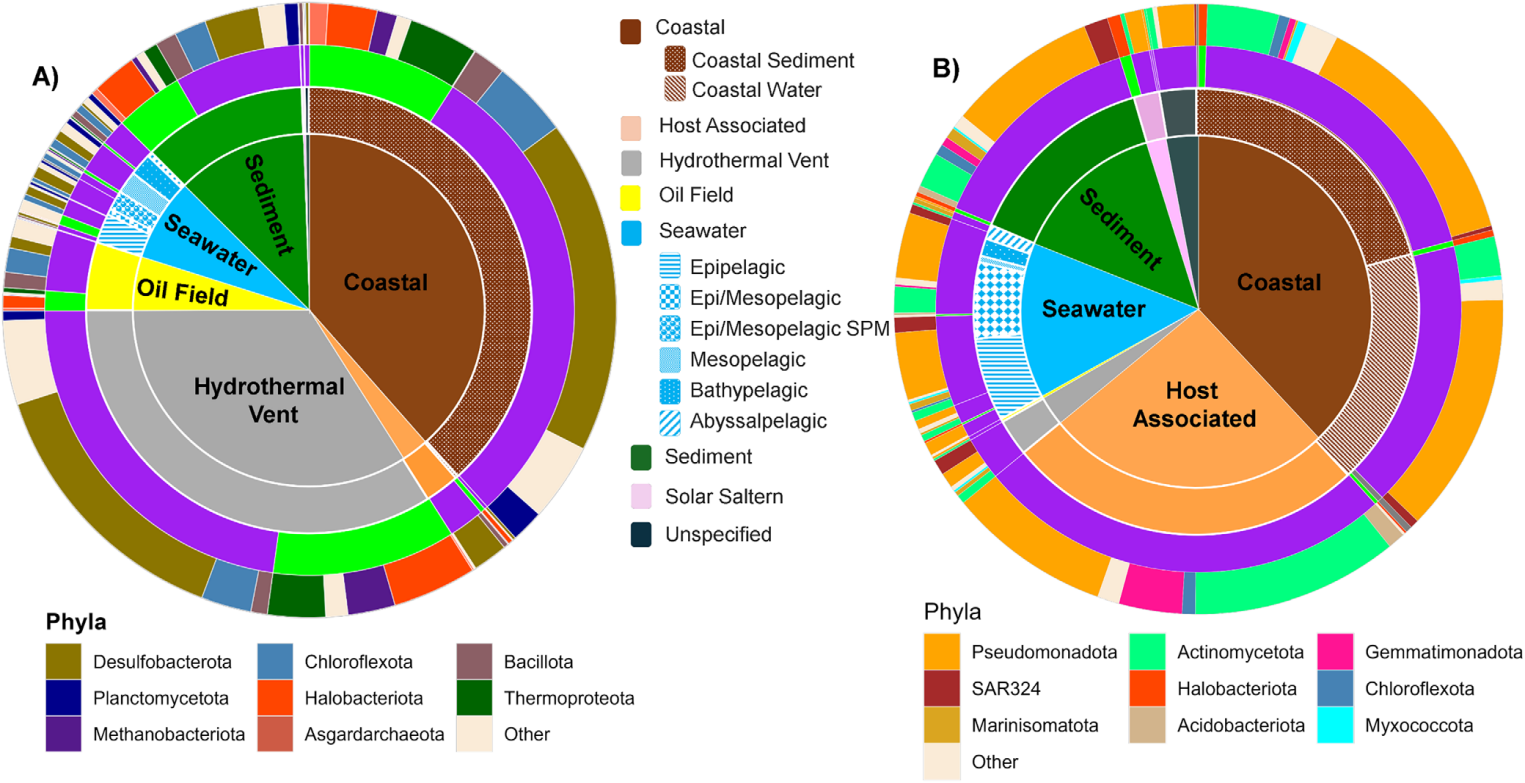
Distribution of (A) Ni‐CODH and (B) Mo‐CODH genes across different oceanic zones. The sunburst plots show the occurrence of the CODH gene across oceanic zones (innermost ring). The second ring provides additional habitat‐level subdivision for the seawater and coastal zones, while other zones remain unpartitioned. The third ring represents the taxonomic domain (*Archaea* in green or *Bacteria* in violet), while the outermost ring indicates the phylum‐level classification of genomes that bear these CODH genes. Phyla representing more than 5% of Ni‐CODH sequences and 1% of Mo‐CODH sequences are highlighted by colour, whereas less represented phyla are grouped as ‘Other’.

To compare how environmental conditions shape the distribution of the two types of CODHs, we grouped the marine‐derived sequences into seven biomes: hydrothermal vents, coastal, seawater, sediment, host‐associated environments, oil fields and solar salterns (Figure 2). The coastal environment was further categorised into water and sediment fractions, and seawater ecosystems were sorted by depth.

Coastal environments represent one of the dominant habitats of both Ni‐ and Mo‐CODH coding genomes, consistent with earlier studies reporting higher rates of microbial CO oxidation in coastal areas (Tolli and Taylor 2005; Yang et al. 2024) (Figure 2). Interestingly, the two enzymes display local niche preferences: Ni‐CODHs showed prevalence in the coastal sediments (Figure 2a), whereas Mo‐CODHs occurred in both coastal waters and sediments (Figure 2b). In contrast, hydrothermal vents stood out as one of the hotspots for Ni‐CODH–encoding genomes (Figure 2a). These deep‐sea systems circulate geothermally heated fluids that are both highly reduced and anoxic, enriched in H_2_, H_2_S, CH_4_ and Fe^2+^ (Brazelton 2017). The dominance of O_2_‐sensitive Ni‐CODHs in vent‐associated communities is therefore expected, as anaerobic CO oxidation can support acetogenesis and methanogenesis, which sustain primary production in the absence of sunlight. Likewise, the oil‐field ecosystems, which are characterised by reducing conditions (Varjani and Gnansounou 2017), exhibited a clear predominance of Ni‐CODHs (Figure 2a).

Across the open‐ocean water column, Mo‐CODHs were detected at all depths but were most abundant in the epipelagic and mesopelagic zones (Figure 2b). Ni‐CODHs, on the other hand, are primarily associated typically with low‐oxygen environments, including particle‐rich waters from the epi‐ and mesopelagic layers of the Black Sea, anoxic basins and deep‐sea brine pools (Figure 2a). In the marine sediments, Mo‐CODHs occurred mainly in oxygen‐exposed surficial and ferromanganese‐rich deposits, whereas Ni‐CODHs were concentrated in reduced subsurface layers such as cold seeps and mud volcanoes found in continental shelves, where anaerobic carbon and sulphur cycling dominate (Figure 2, Data S1 and S2).

Genomes associated with phytoplankton phycospheres (diatoms, algae and dinoflagellates), corals, sponges and marine worms were classified as ‘host‐associated’. Consistent with the predominantly sunlit and oxygenated conditions inhabited by these hosts, most potential CO‐utilisers in this biome encoded Mo‐CODH genes (Figure 2). The importance of CO oxidation in host‐associated systems was demonstrated in the marine gutless worm *Olavius algarvensis* whose bacterial symbionts used CO‐derived energy to support their symbiosis (Kleiner et al. 2012, 2015). We included solar salterns in the marine habitat category, as they are shallow ponds connected to the coastal ocean. The potential CO metabolizers from solar salterns primarily encoded Mo‐CODHs, consistent with their sunlit and oxygen‐rich character (Figure 2). Overall, our analysis shows that potential CO‐utilising genomes are ubiquitous across marine habitats.

At the taxonomic level, Ni‐CODH–encoding microorganisms exhibited broad phylogenetic diversity, spanning 39 bacterial and 10 archaeal phyla, with notable representation from the archaeal phyla *Thermoproteota* and *Halobacteriota*, as well as the bacterial phyla *Desulfobacteriota* and *Chloroflexota* (Figure 2a, Figure S3a, Tables S1 and S2). Mo‐CODHs were largely restricted to bacteria, spanning 14 phyla but dominated by *Pseudomonadota* and *Actinomycetota*; only 19 sequences were recovered from archaea, mainly *Halobacteriota* (Figure 2b, Figure S3b, Tables S3 and S4).

### 3.3 Effect of Oxygen Availability on the Distribution of Ni‐ and Mo‐CODHs in Marine Habitats

The biome‐level analysis described above suggests an ecological separation between the two types of CO dehydrogenases based on oxygen availability. To clearly evaluate this relationship, we examined metagenomic datasets from three marine niches: (i) the seasonally anoxic water column of Saanich Inlet (PRJNA247822), (ii) a methane‐seep system in the South China Sea (PRJNA707313 and PRJNA1115057), and (iii) a 300‐km transect off the San Francisco coast (PRJNA1054206). In all cases, short‐read metagenomic analyses were performed to quantify changes in Ni‐ and Mo‐CODH abundance in response to oxygen gradients (Data S3).

**FIGURE 3:**
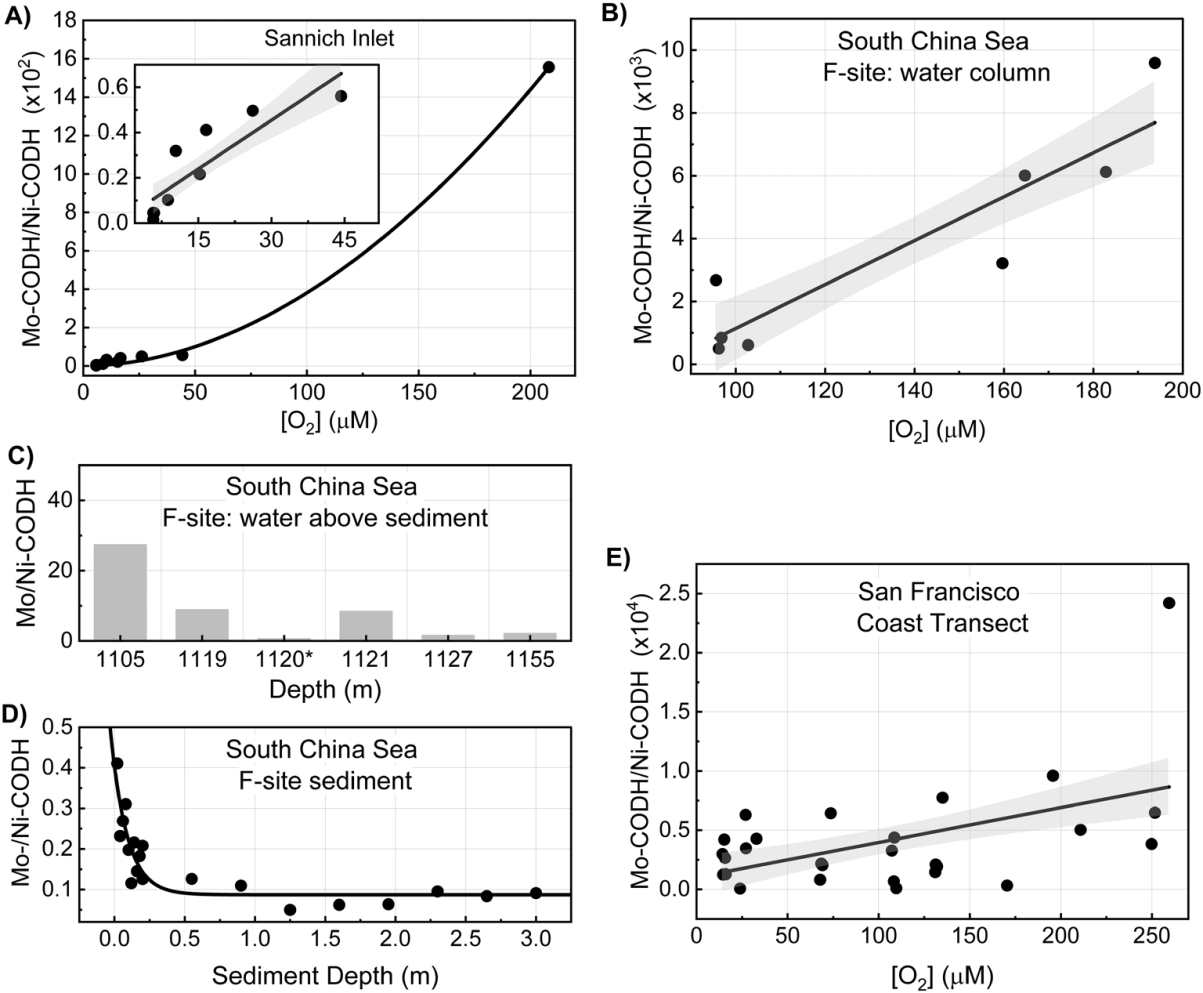
O_2_‐dependent variation in the relative gene abundances (average gene copies per genome) of Mo‐ and Ni‐CODHs across three marine habitats. To evaluate shifts in CODH types along oxygen gradients, the ratio of gene abundances of Mo‐CODH to Ni‐CODH (Mo‐CODH/Ni‐CODH) is plotted against dissolved O_2_ concentration for (A) Saanich Inlet, (B) South China Sea site F water column, and (E) water column samples from a 300 km transect off the San Francisco coast. The inset in panel A shows a magnified view of the ratio at low oxygen concentrations. For environments where dissolved O_2_ metadata were unavailable, the same ratio is plotted as a function of depth for (C) the site F sediment–water interface and (D) site F subsurface sediments, with depth serving as a proxy for oxygen availability. The depth of 1120 m (marked with an asterisk in panel C) corresponds to a zone beneath the invertebrate community characterised by very low oxygen concentrations.

Saanich Inlet is a fjord on the Canadian Pacific coast where a shallow glacial sill restricts deep‐water renewal, leading to vertical stratification and development of hypoxic to anoxic conditions at depth (Torres‐Beltrán et al. 2017). We observed that Mo‐CODH genes were most abundant in the upper water, while Ni‐CODH abundance increased with depth (Figure S4). As a result, the Mo‐CODH/Ni‐CODH gene abundance ratio declined by nearly three orders of magnitude, from ~1500 at 10 m to ~1.5 at 200 m (Figure 3A). This decline reflects a loss of strong Mo‐CODH dominance with depth, driven primarily by increasing Ni‐CODH abundance rather than a pronounced decrease in the abundance of Mo‐CODH genes (Figure S4). The pattern closely tracks the vertical oxygen gradient of the Saanich Inlet (Figure 3A). The narrowing differences between Mo‐ and Ni‐CODH gene abundances at deeper O_2_‐depleted water do not indicate preferential selection for Ni‐CODH under anoxic conditions but instead suggest that Ni‐CODH is unfavoured under oxygenated conditions, while Mo‐CODH is not systematically excluded under low‐oxygen conditions.

In the South China Sea cold seep site F, the oxygen concentration in the overlying water column declined more gradually (from ~200 μM at 30 m to ~100 μM at 1100 m) (Liu et al. 2025). Correspondingly, depth‐related changes in CODH gene abundances within the water column were relatively modest. As O_2_ increased, both Mo‐CODH and Ni‐CODH abundances declined slightly, with Ni‐CODHs showing a marginally stronger decrease (Figure S5), resulting in an approximately 10‐fold reduction in the Mo‐CODH/Ni‐CODH ratio (Figure 3B). However, at the sediment–water interface, a significant shift in the abundances of Ni‐ and Mo‐CODHs was observed. Cold seep sediments are characterised by strong upward fluxes of methane from seep vents, which fuel microbial aerobic methane oxidation at the sediment surface and in the bottom surface water (Fu et al. 2024). This process rapidly consumes oxygen, generating sharply oxygen‐depleted microenvironments even when the overlying water column remains oxygenated. Consistent with these conditions, Ni‐CODH gene abundance increased strongly at the sediment–water interface (Figure S6A), leading to a marked reduction in the Mo‐CODH/Ni‐CODH ratio (Figure 3C). Within subsurface sediments, where oxygen penetration is typically restricted to only the upper few centimetres (Boetius and Wenzhöfer 2013), this ratio declined by an additional ~5‐fold (Figure 3D), reflecting the increasing dominance of anaerobic CO oxidation (Figure S6B). Notably, the dominant Ni‐CODH type also changed from bacterial *cooS*‐type Ni‐CODHs, which prevailed in the water column, to archaeal *cdhA*‐type Ni‐CODHs, becoming dominant below a sediment depth of ~50 cm (Figure S6C). This shift is consistent with the well‐established enrichment of archaeal lineages in cold seep sediments (Wang et al. 2025).

In the third system, a 300‐km transect off the coast of San Francisco, the dissolved oxygen profile does not exhibit a simple depth profile (Arandia‐Gorostidi et al. 2024). The oxygen concentration decreases from ~260 μM at 50 m to ~15 μM at 1000 m, then rises again to ~130 μM at 4000 m (Arandia‐Gorostidi et al. 2024). Correspondingly, CODH patterns followed a complex depth variation. Mo‐CODH abundance increased moderately with depth (~5‐fold), and Ni‐CODH abundance increased by ~100‐fold down to 2000 m before declining slightly in deeper waters (Figure S7A,C). With the decrease in oxygen concentration, both Mo‐ and Ni‐CODHs abundances increased, with the rate of Ni‐CODH increasing slightly more (Figure S7B,D). As a result, the Mo‐CODH/Ni‐CODH ratio declined marginally with the decrease in oxygen concentration (Figure 3E).

Taken together, these three systems demonstrate that oxygen availability is a critical environmental driver shaping the balance between the two CODH types. The changes in their relative gene abundances along oxygen gradients are primarily driven by the absence of Ni‐CODHs in oxic environments, rather than by a loss of Mo‐CODHs under oxygen‐limited conditions. The gene abundance of Mo‐CODHs shows more complex and system‐specific relationships with oxygen concentration and is likely influenced by additional environmental factors, such as metal (iron) availability, as suggested by Lappan et al. (2023).

### 3.4 Phylogenetic and Structural Diversity of Marine Ni‐CODHs

**FIGURE 4:**
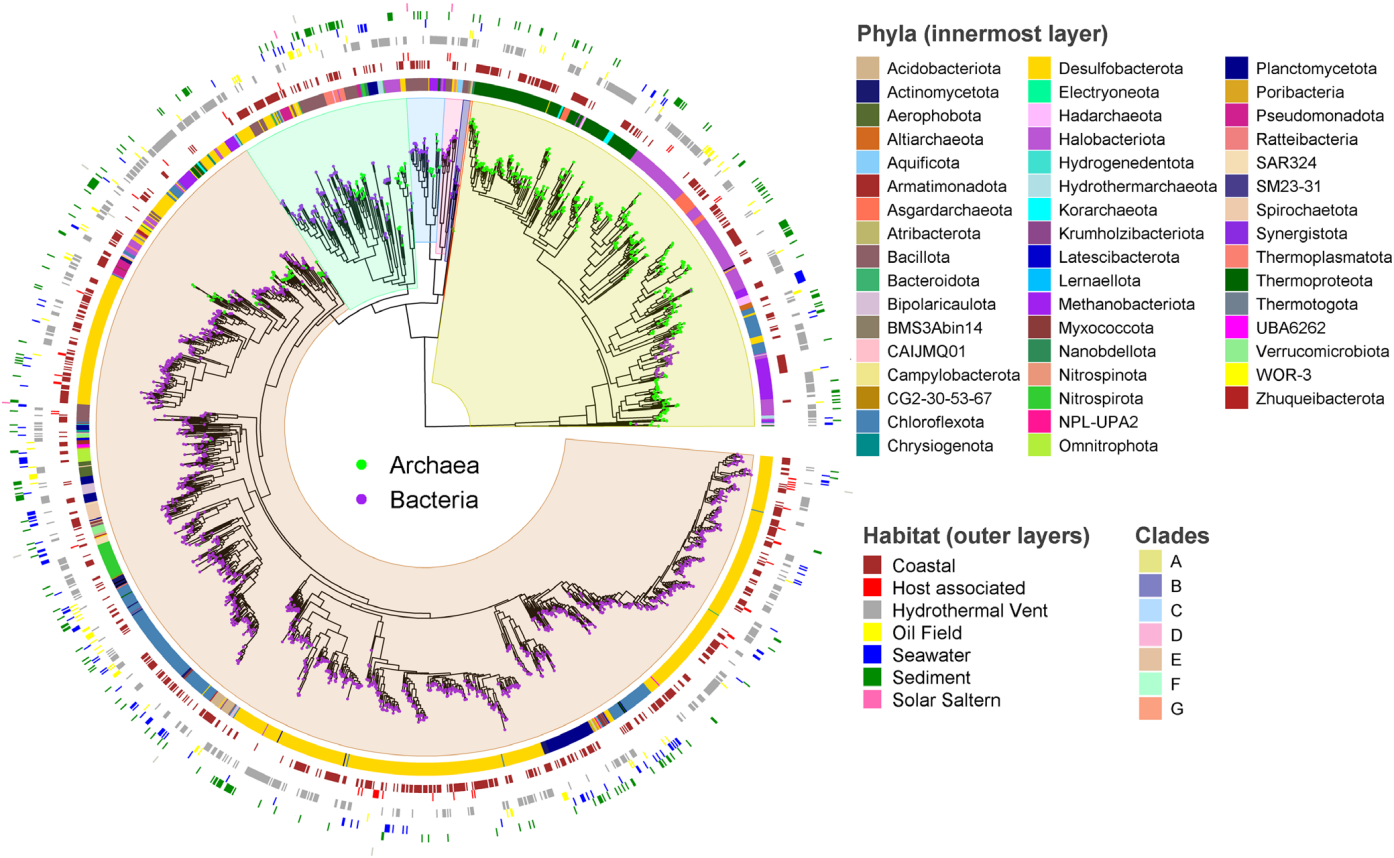
Maximum‐likelihood‐based phylogenetic tree of Ni‐CODH sequences from marine prokaryotes. The clades (A–G) of the tree are highlighted using the colour scheme shown in the legend. The tips of the tree are coloured according to the kingdom of the Ni‐CODH source organism. The innermost ring represents the phyla of the prokaryotes that contain these Ni‐CODH genes. The seven outer rings show the isolation source information of the genomes.

A maximum‐likelihood‐based tree was constructed using 1969 marine Ni‐CODH sequences identified in this study to examine their phylogenetic diversity (Figure 4). The sequences were grouped into seven previously defined clades (A–G; Techtmann et al. 2012; Inoue et al. 2019), all supported by high bootstrap values (> 0.9; Figure S8). The majority of sequences (~65%) fell within clade E, followed by clades A (23%) and F (8%) (Figure 4). Clades B, C, D and G accounted for only ~4% of the sequences in the dataset (Figure 4). Around 20% of marine genomes identified here contain more than one Ni‐CODH gene. While Ni‐CODHs from clades B, C and D occurred only once in the genomes, those from clades A, E and F co‐occurred within the same genome (Figure S9A). Co‐occurrence analysis across different clades showed generally limited co‐distribution, with a few moderate and asymmetric associations. For instance, clade B was frequently detected alongside clade F, clade C with clade A and clades D and F with clade E, whereas the reverse relationships were much less common (Figure S9B). This asymmetry likely reflects differences in clade representation within the dataset, with less‐represented clades more frequently detected alongside more prevalent ones. Overall, co‐occurrence patterns within and between clades are broadly consistent with those reported by Böhm and Land (2026). A prominent feature of the phylogeny is the sharp division between the CdhA‐type and CooS‐type Ni‐CODHs. All CdhA‐type sequences clustered exclusively in clade A, while clades B–G consisted solely of CooS‐type homologues (Figure 4), in agreement with earlier analyses (Techtmann et al. 2012; Inoue et al. 2019). This deep split reflects extensive structural and sequence differences between the two types, including CdhA‐specific insertions that accommodate additional Fe‐S clusters, a C‐terminal extension and a low global sequence identity apart from the C‐cluster ligating residues (Figure S10) (Techtmann et al. 2012). As CdhA‐type Ni‐CODHs are largely found in archaea, clade‐A was dominated by members of *Thermoproteota*, *Halobacteriota* and *Methanobacteriota* (Figure 4, Figure S11; Data S1). A small number of sequences from the bacterial phyla *Chloroflexota* and *Desulfobacteriota* also appeared in clade A, suggesting that these organisms encode archaeal‐type CdhA homologues—an observation also reported by Inoue et al. (2019). In contrast, the remaining clades were largely composed of bacterial Ni‐CODHs and did not display a strong phylum‐level structuring (Figure 4, Figure S11). The scattered distribution of clades across bacterial and archaeal lineages, characterised by broad taxonomic dispersion and the coexistence of distinct clades within individual phyla, is consistent with previous proposals that Ni‐CODHs underwent extensive horizontal gene transfer and gene loss during evolution (Techtmann et al. 2012).

Analysis of sequence motifs binding the accessory Fe▬S B‐ and D‐clusters showed that cysteines coordinating the B‐clusters were strictly conserved across all Ni‐CODHs, whereas the D‐cluster–ligating cysteines were variable and even absent in some cases. Based on the number and amino acid spacing the D‐cluster–ligating cysteines, Ni‐CODHs were classified into five types: type I (Cys‐x_3_‐Cys), type II (Cys‐x_7–16_‐Cys), type III (Cys‐x_2_‐Cys), type IV (single cysteine) and type V (no cysteines) (Figure S12). The crystal structures of types I, II and III D‐cluster‐containing Ni‐CODHs are available, which revealed that types I and II coordinate a [4Fe‐4S] cluster, the type II D‐cluster is [2Fe‐2S] type (Gong et al. 2008; Dobbek et al. 2004; Wittenborn et al. 2018) (Figure S12). Notably, the presence of a type II D‐cluster has been linked to increased O_2_ tolerance of *Desulfovibrio vulgaris* Ni‐CODHs (Wittenborn et al. 2020). When mapped onto the phylogenetic tree, D‐cluster types II and III were broadly distributed across clades B–E, whereas type I was restricted to clade A (Figure S13). Type V D‐cluster–containing Ni‐CODHs were found in clade A and in a small number of archaeal sequences within clade F (Figure S13). Type IV Ni‐CODHs were rare (*n* = 17) and occurred primarily in archaeal sequences within clade C, as well as in clade A (Figure S13). Overall, the differentiation of D‐cluster architectures appears to correlate with taxonomic lineage, distinguishing bacterial and archaeal Ni‐CODHs, and indicating that D‐cluster composition is largely constrained by early divergence between these enzyme types. Differences in D‐cluster architecture may influence electron transfer from the catalytic C‐cluster to downstream redox partners.

To assess whether Ni‐CODH phylogeny is linked to environmental origin, we mapped habitat information onto the phylogeny (Figure S14). No clades showed habitat‐specificity, as Ni‐CODH sequences from diverse marine environments were interspersed throughout the tree. The absence of habitat‐specific clustering indicates that the phylogenetic diversification of marine Ni‐CODHs is largely independent of the environmental conditions in which their host genomes reside.

### 3.5 Function Prediction of Ni‐CODHs Based on Their Genomic Context Analysis

**FIGURE 5:**
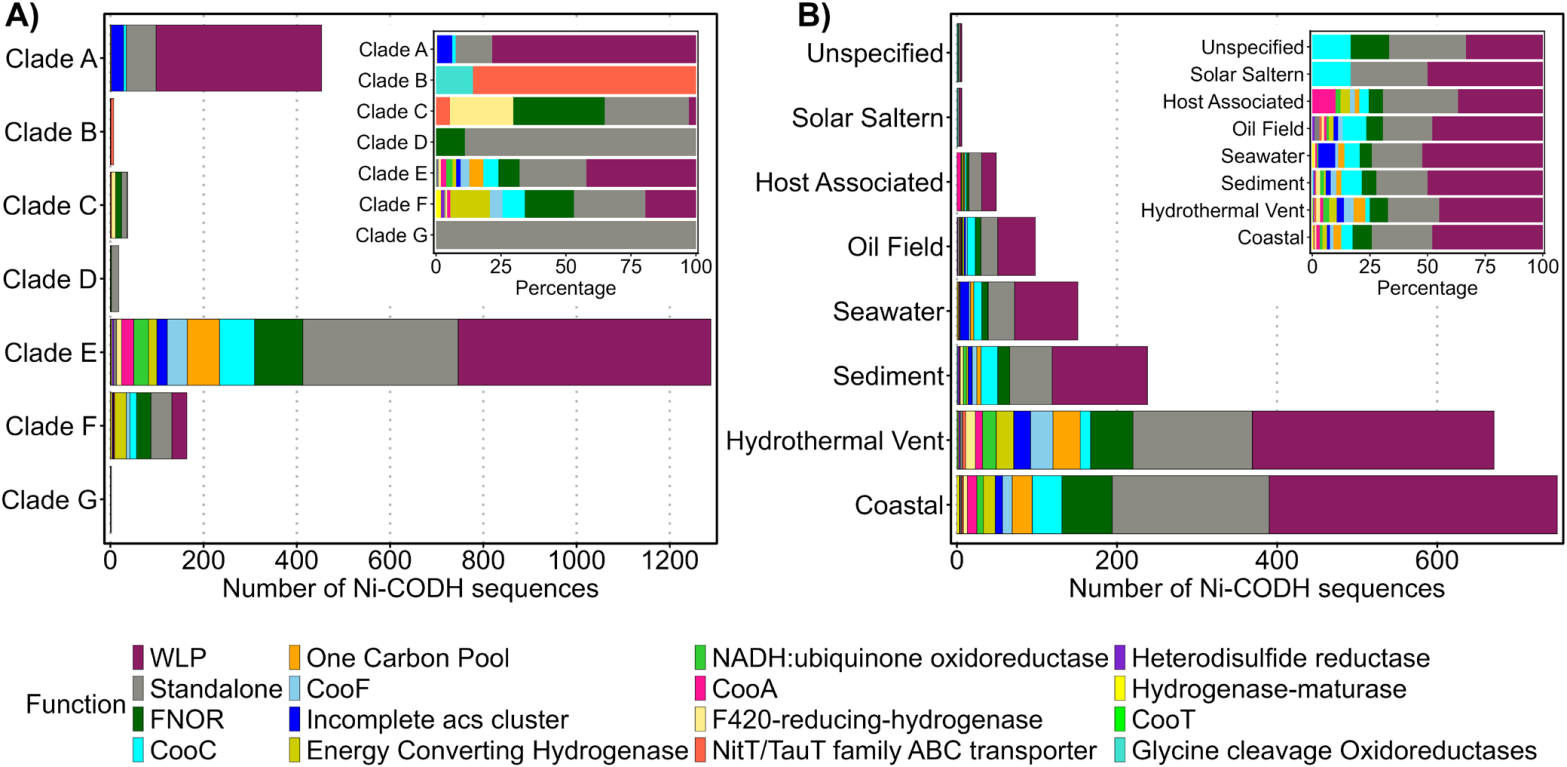
Distribution of Ni‐CODH genome contexts across the phylogenetic clades and marine environments (A) The clade‐wise composition of genome context‐based functional information of Ni‐CODHs. (B) Distribution of the same genome‐context information across different oceanic locations. The insets in both panels indicate the percentage representation of the same result.

As Ni‐CODH genes often occur adjacent to their functional partners, genomic neighbourhood analysis was used to predict their physiological roles (Techtmann et al. 2012; Adam et al. 2018; Inoue et al. 2019). Applying this methodology, we classified marine Ni‐CODHs into 15 functional groups, based on the identity of the proteins encoded by proximal genes (±15 genes from *coos* or *cdhA*) (Figure 5, Figure S15; Table S5). About 24% of all sequences lacked any known CO‐metabolism genes and were labelled as ‘standalone’ (Figure 5). Many of the multiple Ni‐CODH encoding marine genomes had them in different genomic contexts, including standalone variants. These genomes could employ diverse CO‐dependent metabolic pathways in response to specific environmental conditions. However, experimental information on whether these genetic differences correspond to distinct physiological roles under specific environmental conditions is lacking.

Clade‐A Ni‐CODHs, which are the typical archaeal CdhA‐type enzymes, display the most consistent genome neighbourhood patterns (Figure 5A). Most of these Ni‐CODHs were adjacent to the *cdhC* gene, encoding the β‐subunit of the archaeal ACS complex that catalyses acetyl‐CoA formation in the carbonyl branch of the WL pathway (Figure 5A, Figure S16) (Can et al. 2014), hence classified into ‘WLP’ functional group. In several cases, the complete WLP gene cluster, which includes additional ACS subunit genes (*cdh*BDE, encoding the methyl‐transfer and electron transfer components of the archaeal ACS complex), is also present in the Ni‐CODH neighbourhood (Figure S16, Data S1) (Adam et al. 2018).

Notably, some genomes, including that of the hydrothermal vent archaeon *Archaeoglobus fulgidus* , have the clade‐A Ni‐CODH genes adjacent to *cdh*BDE genes, whereas the *cdh*C gene is located at a distant genomic locus (Data S1). Experimental evidence showed that *A. fulgidus* can grow chemolithoautotrophically on CO (Henstra et al. 2007). Thus, these Ni‐CODHs were also assigned to the ‘WLP’ category. A small subset of clade‐A Ni‐CODH‐containing genomes lacked the *cdhC* gene entirely but contained other *cdh* genes in proximity; thus, they were designated as ‘incomplete acs cluster’ (Figure 5A, Data S4).

Clade B CODHs were mainly associated with ATP‐binding cassette (ABC) transporter genes or, less often, with glycine cleavage genes (Figure 5A). As the specific involvement of these proteins with CO metabolism is unclear, the physiological role of clade B Ni‐CODHs remains unknown. Clade C bacterial Ni‐CODHs often neighbored genes like FAD‐NAD(P) oxidoreductase (FNOR) and ferredoxin‐like proteins (CooF) (Figure 5A). These associated FNOR and CooF proteins were hypothesised to direct electrons from CO oxidation towards energy conservation or help mitigate oxidative stress by detoxifying reactive oxygen species (ROS) (Wu et al. 2005; Geelhoed et al. 2016). The archaeal Ni‐CODHs in clade C are primarily found with genes for F_420_‐reducing hydrogenases, which are crucial for methanogenesis (Hendrickson and Leigh 2008). Many of these genomes harbour distant *cdhC* genes, although their functional consequence remains unclear. Clade‐D contains 18 bacterial Ni‐CODH sequences, of which 16 are ‘standalone’, and two are present alongside FNOR and CooF genes (Figure 5A, Data S1).

Clade E, the largest and most metabolically diverse group, included Ni‐CODHs from almost all functional categories (Figure 5A). Approximately 42% of them were classified into the ‘WLP’ group because they had the a*csβ* gene (corresponding to the *cdhC* gene of archaea) in the proximity (Figure 5A, Figure S15). In several genomes, *acsβ* was located distantly, but Ni‐CODHs were colocalized with other ACS subunit genes; these were likewise assigned as ‘WLP’ (Data S1). The clade‐E Ni‐CODHs associated with genes expressing enzymes associated with the one‐carbon pool, such as formate dehydrogenase (*fdh*), formyl‐tetrahydrofolate synthase (*fts*), methylene‐tetrahydrofolate dehydrogenase (*folD*) and methylene‐tetrahydrofolate reductase (*metF*), were designated as ‘One Carbon Pool’ (Figure 5A, Data S1).

Other clade E members were either standalone CODHs or linked to various metabolic partner genes, including FNOR–CooF clusters, CooC metallochaperones, CooA regulators, NADH: ubiquinone oxidoreductases, heterodisulphide reductase and energy‐converting [NiFe] hydrogenases (ECH) (Figure 5A). ECH‐associated Ni‐CODHs were especially notable in thermophilic *Thermococcus* species from deep‐sea vents, where CO oxidation supports hydrogen or sulphide production, independent of the WL pathway (Kozhevnikova et al. 2016). In these organisms, electrons from CODH‐mediated CO oxidation are transferred to the ECH complex, reducing protons to hydrogen and generating an ion gradient that drives ATP synthesis (Figure S17) (Schoelmerich and Müller 2020; Hedderich and Forzi 2005). Notably, across multiple genomic contexts within this clade, *acsβ* genes were frequently present but located distantly from the Ni‐CODH locus (Figure S18 and Data S4).

Although numerically smaller than clade E, clade F also displayed substantial functional diversity, encompassing all major categories identified in this study. Most clade F Ni‐CODHs were distributed among the WLP (19%), FNOR (18%) and ECH (15%) contexts, underscoring the metabolic versatility of this clade (Figure 5A). Clade G consisted of only two Ni‐CODH sequences, both of which are standalone. Notably, across all clades, approximately 21% of genomes lacking Ni‐CODHs in the WLP context contained *cdhA/acsB* genes elsewhere in the genome (Figure S18).

Across all clades, the relationship between genomic context and phylogeny was weak. Similar operon structures appeared in multiple clades, while closely related sequences often differed in their gene neighbourhoods (Figure 5A and Figure S15). This pattern suggests a complex evolutionary history driven by extensive horizontal gene transfer and genome rearrangements, consistent with earlier research (Techtmann et al. 2012). We also investigated whether specific genomic contexts were more common in certain marine habitats. However, there were no environment‐specific biases of the genome contexts (Figure 5B). Overall, these findings indicate that marine Ni‐CODHs, despite originating from a limited portion of global microbial diversity, encode the full range of known anaerobic CO‐metabolism pathways.

### 3.6 Phylogenetic Analysis of Mo‐CODHs of Marine Source

**FIGURE 6:**
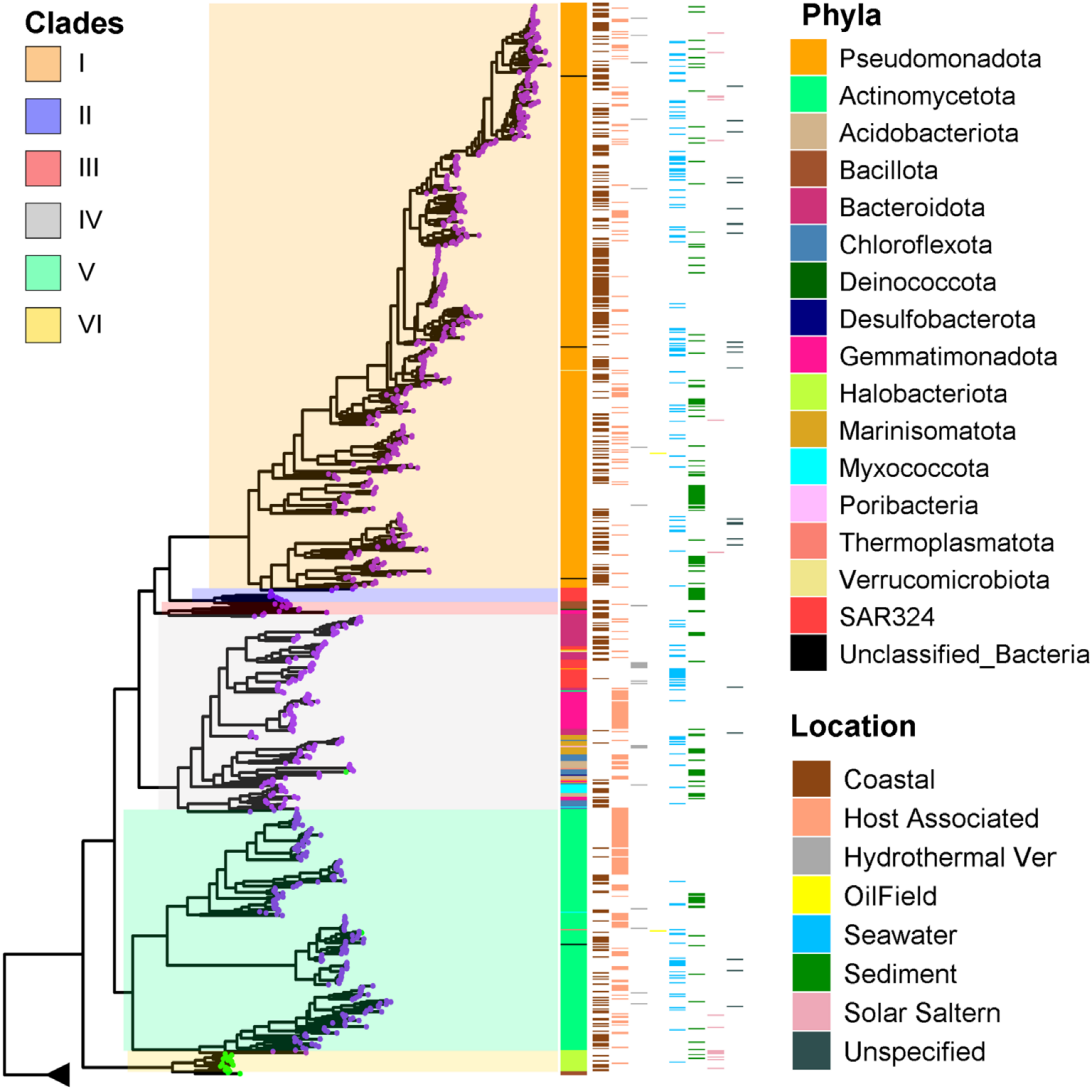
A maximum‐likelihood phylogenetic tree was constructed using 864 marine Mo‐CODH sequences. The root node, composed of three form II CODHs, is collapsed and shown as a triangle. The tips are colour‐coded by the kingdom of the source organism (Archaea in green and Bacteria in violet) and divided into six clades (I–VI) according to the legend. The innermost layer displays the phyla of the Mo‐CODH genomes, while the eight outer layers indicate the isolation sources. An unspecified location implies that the genome is of marine origin but lacks specific location information.

To assess the phylogenetic diversity of marine Mo‐CODHs, we constructed a maximum‐likelihood phylogeny using 864 form I CoxL sequences, with three form II CODHs included as outgroups. The resulting tree resolved six well‐supported clades (Figure 6 and Figure S19), with four clades exhibiting clear coherence at the phylum level (Figure 6; Figure S20A). Clade I was dominated by sequences from *Pseudomonadota*, Clade II comprised *SAR324*‐affiliated Mo‐CODHs, Clade V was enriched in *Actinomycetota*, and Clade VI consisted exclusively of archaeal Mo‐CODHs from *Halobacteriota*. In contrast, clades III and IV were phylogenetically more heterogeneous, containing Mo‐CODHs from multiple bacterial phyla, including *Bacteroidota*, *Bacillota*, *Gemmatimonadota* and others (Figure 6; Figure S20A).

Mo‐CODH‐encoding archaea were rare in our dataset. Most of these archaeal sequences clustered within Clade VI; however, two additional archaeal Mo‐CODHs from the phylum *Thermoplasmatota* were detected in Clades III and IV, respectively, which were otherwise dominated by bacterial sequences. Mo‐CODHs are scarce in archaea across various environments, with only a few halophilic and thermophilic representatives reported (G. M. King 2015; McDuff et al. 2016; Sokolova et al. 2017). It has been proposed that archaea acquired Mo‐CODHs from bacteria relatively late in evolutionary history, potentially following the rise of atmospheric oxygen (Sokolova et al. 2017).

The occurrence of both phylum‐coherent and taxonomically mixed clades indicates that Mo‐CODH phylogenetic relationships do not consistently correspond to host phylum‐level classification. Similar clustering patterns have been reported in global surveys of Mo‐CODHs across the full biosphere (Cordero et al. 2019). Like Ni‐CODHs, phylogenetic clades of Mo‐CODHs also did not show habitat preference (Figure 6, Figure S20B).

Form I Mo‐CODHs are typically encoded by operons containing genes for the small (*coxS*), medium (*coxM*) and large (*coxL*) subunits arranged in the order *coxMSL* (King and Weber 2007). Consistent with this organisation, 810 of the 864 marine Mo‐CODHs identified here were associated with complete *coxMSL* operons. The remaining sequences, primarily derived from MAGs, contained partial operons (*coxSL* or *coxL* alone), likely reflecting incomplete genome recovery rather than true loss of subunits.

Although aerobic CO oxidation alone does not typically sustain microbial growth, Mo‐CODHs can supply a low‐level energy yield that may support survival under nutrient‐limiting conditions (Cordero et al. 2019). Electrons released during CO oxidation are transferred to the aerobic respiratory chain via the quinone pool (Wilcoxen et al. 2011; Greening and Grinter 2022). The menaquinone‐binding membrane protein CoxG was proposed to deliver electrons from CO oxidation to the quinone pool (Pelzmann et al. 2014; Kropp et al. 2025). In our dataset, coxG genes were identified within the coxL genomic context (±15 genes) in 573 of 864 CoxL‐containing genomes (~66%; Data S1). Among the remaining genomes, 235 (~27%) encoded coxG homologues elsewhere in the genome (Data S1). Thus, 806 of 864 genomes (93%) harbour coxG homologues, either within operons or at distant genomic locations. These results suggest that CoxG‐mediated electron transfer to the quinone pool is a widespread feature of marine Mo‐CODH systems, potentially supporting supplementary energy generation under nutrient‐limited conditions.

## 4 Discussion

This study provides a broad genome‐wide survey of CODH distribution across marine habitats. Previous studies have typically focused on either Ni‐CODHs or Mo‐CODHs separately without examining their combined distribution within a single ecosystem (Inoue et al. 2019; Cordero et al. 2019). Katayama et al. recently investigated CODH‐mediated metabolism in the human gut, but could not detect Mo‐CODHs in that system, thereby precluding a comparative analysis of the two enzyme types (Katayama et al. 2024). More recently, Latorre et al. investigated the abundance of both Ni‐ and Mo‐CODHs in volcanic ecosystems using MAGs derived from metagenomic datasets (Latorre et al. 2026). In contrast, we performed large‐scale database mining of both isolate and metagenomically assembled genomes to identify Ni‐ and Mo‐CODH sequences of marine origin. Subsequent phylogenetic reconstruction and genomic context analyses revealed their evolutionary diversity and potential functional roles within the marine ecosystem. Furthermore, short‐read metagenomic abundance profiling was conducted to examine the ecological preferences of these two CODH types.

Our results show that both Ni‐CODH and Mo‐CODH genes are widely distributed across marine lineages spanning 53 microbial phyla and occur in diverse marine habitats. This ubiquitous distribution of CODHs across marine niches is consistent with previous reports identifying microbial CO oxidation as the dominant sink for oceanic CO (Tolli and Taylor 2005; Conte et al. 2019; Greening and Grinter 2022). Notably, coastal environments showed the highest representation of both CODH types. This pattern may reflect elevated biological CO‐oxidation in coastal systems, where CO concentrations are typically higher due to intense photochemical production (Yang et al. 2024). However, further experimental evidence is required to substantiate this association.

Metagenomic short‐read analyses from three distinct marine niches (Saanich Inlet, the South China Sea cold seep water column and sediments, and water columns from transects off the San Francisco coast) showed that the Mo‐CODH/Ni‐CODH gene ratio generally decreased with declining oxygen availability (Figure 3). This decrease was driven primarily by an increase in Ni‐CODH abundance under oxygen‐limited conditions rather than by a substantial decrease in Mo‐CODH abundance. Mo‐CODHs were not strongly excluded under oxygen‐limited conditions, as reflected by their substantial abundance across a broad range of oxygen gradients. In addition to oxygen, Mo‐CODHs may also couple CO oxidation to alternative electron acceptors such as nitrate under low‐oxygen conditions (G. M. King 2006; Imaura et al. 2023). Therefore, it is unsurprising that Mo‐CODH abundance does not always correlate with oxygen availability and may be influenced by multiple environmental factors. In this context, Lappan et al. suggested that an increase in Mo‐CODH gene abundance with depth in the marine water column could be associated with elevated metal (iron) concentrations and the increased need for an alternative to phototrophic energy‐harvesting mechanisms in deeper aphotic waters, where phototrophy is limited (Lappan et al. 2023). Overall, these observations suggest that oxygen primarily exerts negative selection on Ni‐CODHs, thereby limiting their prevalence in oxic marine environments.

Phylogenetic analysis of Ni‐CODHs indicates that all previously described clades (A–F) are represented in the marine ecosphere, with most belonging to clades A, E and F (Figure 4). This pattern is consistent with a previous biome‐scale analysis, which identified these clades as dominant Ni‐CODH groups in aquatic environments (Inoue et al. 2022), and differs from host‐associated systems, including the human gut, where Ni‐CODHs are enriched in clades B–E with lower representation of clades A and F (Inoue et al. 2022; Katayama et al. 2024). Notably, clade distributions remain broadly consistent within marine sub‐environments, suggesting that the phylogenetic composition of Ni‐CODHs may be shaped by broad ecological categories rather than variation within sub‐biomes of a given habitat. Phylogenetic analysis of marine Mo‐CODHs similarly reveals their diversity in the ocean (Figure 6), indicating substantial evolutionary diversity of these enzymes in this environment.

Genome context analysis showed that clade A Ni‐CODHs are frequently associated with genes encoding components of the WL pathway, whereas those from clades E and F occur in more diverse genomic neighbourhoods, including genes of the ACS complex, other one‐carbon metabolic processes, energy‐converting [NiFe] hydrogenases and additional oxidoreductases. In contrast, Ni‐CODHs from clades B and D are often found as standalone genes or in association with genes whose roles in CO metabolism are poorly understood. Clade C Ni‐CODHs are commonly found in the vicinity of FAD–NAD(P) oxidoreductases or F_420_‐reducing hydrogenases, although their functional roles remain unclear. These clade‐specific gene‐association patterns are consistent with the findings of Böhm and Land (2026), who, based on operon architecture, proposed that Ni‐CODHs from clades A, E and F are more likely to efficiently catalyse CO–CO_2_ interconversion (Böhm and Land 2026). The predominance of these clades in marine environments may therefore be a consequence of the functional advantages provided by these operon architectures. Overall, marine Ni‐CODHs likely perform diverse roles, including carbon fixation, energy conservation, redox balancing and reactive oxygen species detoxification under oxygen‐limited conditions. Notably, the genomic context of Ni‐CODHs does not appear to be strongly habitat‐specific, indicating that their functional repertoire is broadly conserved across marine environments.

In Mo‐CODH–containing marine genomes, approximately 93% encode coxG, with 66% located adjacent to coxL and 27% elsewhere in the genome. CoxG encodes a quinone‐binding membrane protein that mediates electron transfer from CoxL to the respiratory chain (Kropp et al. 2025). Although the biochemical pathways to which Mo‐CODHs contribute are unclear, it is presumed to provide supplementary energy through electron transfer via the CoxG protein to the respiratory chain under nutrient limitation (Cordero et al. 2019; King and Weber 2007). Thus, this gene‐context analysis supports a role for Mo‐CODHs in low‐level energy acquisition under carbon‐limited conditions.

Taken together, our results demonstrate that CODH‐encoding microbes are widespread across diverse marine ecosystems. Oxygen‐sensitive Ni‐CODHs are underrepresented in oxygen‐rich environments, whereas Mo‐CODHs occur across both oxic and oxygen‐limited niches, indicating their complementary roles in CO oxidation across marine redox gradients. This distribution likely supports a continuous CO‐to‐CO_2_ turnover and constrains ocean‐to‐atmosphere CO flux. Future studies integrating metagenomics, metatranscriptomics, metaproteomics and direct CO oxidation rate measurements across diverse ocean settings will be essential to determine how environmental factors, including oxygen, metal and organic carbon availability, shape the ecological distribution and expression of these enzymes.

## Author Contributions

**Nipa Chongdar:** conceptualization, investigation, funding acquisition, writing – original draft, methodology, validation, visualization, writing – review and editing, software, formal analysis, project administration, data curation, supervision, resources. **Anand Goyal:** data curation, writing – review and editing. **Samir R. Damare:** writing – review and editing, project administration.

## Funding

This work was supported by Department of Science and Technology, Govenrnment of India, DST INSPIRE Faculty Fellowship (DST/INSPIRE/04/2021/002518).

## Conflicts of Interest

The authors declare no conflicts of interest.

## Supporting information


**Table S1:** Ocean zone‐wise distribution of Ni‐CODHs in different archaeal phyla.
**Table S2:** Ocean zone‐wise distribution of Ni‐CODHs in different bacterial phyla.
**Table S3:** Ocean zone‐wise distribution of MoCu‐CODHs in different phyla of Archaea.
**Table S4:** Ocean zone‐wise distribution of MoCu‐CODHs in different phyla.
**Table S5:** Functional categorisation of Ni‐CODHs based on their genome context.
**Figure S1:** Crystal structures of anaerobic and aerobic CODH.
**Figure S2:** Workflow for retrieval and curation of Ni‐CODH and Mo‐CODH datasets.
**Figure S3:** Habitat‐wise composition of the phyla encoding (A) Ni‐CODH and (B) MoCu‐CODH genes in the marine ecosystems.
**Figure S4:** Variation in the average copy numbers of Mo‐CODH and Ni‐CODH genes along depth and oxygen gradients in Saanich Inlet.
**Figure S5:** Variation in the average copy numbers of Mo‐CODH and Ni‐CODH genes along depth and oxygen gradients in *cold‐seep water column* with respect to increasing depth (A), and oxygen (B). Changes in average copy numbers of Ni‐CODH genes with depth (C) and oxygen (D).
**Figure S6:** Variation in the average copy numbers of Mo‐CODH and Ni‐CODH genes in the sediment water interface and within the subsurface sediment of cold‐seep site F.
**Figure S7:** Variation in the average copy numbers of Mo‐CODH and Ni‐CODH genes along depth and oxygen gradients across the San Francisco coastal transect.
**Figure S8:** Phylogenetic tree of marine Ni‐CODH sequences. The bootstrap values (> 0.95) are marked with round labels.
**Figure S9:** Distribution and co‐occurrence of marine Ni‐CODH clades across genomes.
**Figure S10:** Structural comparison of bacterial (CooS) and archaeal (CdhA) Ni‐CODHs.
**Figure S11:** Clade‐wise composition of the phyla coding Ni‐CODH genes. The inset shows the same information in percentage format.
**Figure S12:** Sequence logos and structures of different types of D‐clusters observed in our dataset.
**Figure S13:** The ML‐based phylogenetic tree of Ni‐CODH is mapped with the D‐cluster type information.
**Figure S14:** Distribution of Ni‐CODHs across phylogenetic clades and marine environments.
**Figure S15:** The phylogenetic tree of Ni‐CODHs with genome context information mapped onto the layer bordering the tree.
**Figure S16:** Schemes showing WL‐pathway reactions in hydrogenotrophic methanogens and acetogens.
**Figure S17:** Operon structure and schematic representation of the subunit architechture of a typical Energy converting hydrogenase neighboring a Ni‐CODH gene.
**Figure S18:** Genomic contexts of Ni‐CODH in acsB/cdhC‐gene containing, non‐WLP genomes.
**Figure S19:** Phylogenetic tree of marine MoCu‐CODH sequences. The bootstrap values (> 0.90) are marked with round labels.
**Figure S20:** Phylum‐level taxonomic composition and environmental distribution of marine Mo‐CODH phylogenetic clades.

**Data S1:** Ni‐CODH sequence identifiers, protein names, assembly identifiers, taxonomic details, isolation source and genetic context.

**Data S2:** Mo‐CODH sequence identifiers, protein names, assembly identifiers, taxonomic details, isolation source and genetic context.

**Data S3:** Reads Per Kilobase per Million reads (RPKM) data of Ni‐ and Mo‐CODHs obtained from short read metagenomic analysis and source data of Figure 3.

**Data S4:** Sequence identifiers of AcsB that are not found in the gene context of Ni‐CODHs but are present in the genomes.

## Data Availability

The data that support the findings of this study are available in the Supporting Information of this article.
