# Supplementary material for: Genomic Survey of Carbon Monoxide Dehydrogenases Reveals Their Widespread Distribution in Marine Habitats
Source: Environ Microbiol Rep. 2026 Jun 3;18(3):e70375. doi: 10.1111/1758-2229.70375 (PMC13240472; doi:10.1111/1758-2229.70375)
Supplement: Supplementary file 1 — Table S1: Ocean zone‐wise distribution of Ni‐CODHs in different archaeal phyla. Table S2: Ocean zone‐wise distribution of Ni‐CODHs in different bacterial phyla. Table S3: Ocean zone‐wise distribution of MoCu‐CODHs in different phyla of Archaea. Table S4: Ocean zone‐wise distribution of MoCu‐CODHs in different phyla. Table S5: Functional categorisation of Ni‐CODHs based on their genome context. Figure S1: Crystal structures of anaerobic and aerobic CODH. Figure S2: Workflow for retrieval and curation of Ni‐CODH and Mo‐CODH datasets. Figure S3: Habitat‐wise composition of the phyla encoding (A) Ni‐CODH and (B) MoCu‐CODH genes in the marine ecosystems. Figure S4: Variation in the average copy numbers of Mo‐CODH and Ni‐CODH genes along depth and oxygen gradients in Saanich Inlet. Figure S5: Variation in the average copy numbers of Mo‐CODH and Ni‐CODH genes along depth and oxygen gradients in cold‐seep water column with respect to increasing depth (A), and oxygen (B). Changes in average copy numbers of Ni‐CODH genes with depth (C) and oxygen (D). Figure S6: Variation in the average copy numbers of Mo‐CODH and Ni‐CODH genes in the sediment water interface and within the subsurface sediment of cold‐seep site F. Figure S7: Variation in the average copy numbers of Mo‐CODH and Ni‐CODH genes along depth and oxygen gradients across the San Francisco coastal transect. Figure S8: Phylogenetic tree of marine Ni‐CODH sequences. The bootstrap values (> 0.95) are marked with round labels. Figure S9: Distribution and co‐occurrence of marine Ni‐CODH clades across genomes. Figure S10: Structural comparison of bacterial (CooS) and archaeal (CdhA) Ni‐CODHs. Figure S11: Clade‐wise composition of the phyla coding Ni‐CODH genes. The inset shows the same information in percentage format. Figure S12: Sequence logos and structures of different types of D‐clusters observed in our dataset. Figure S13: The ML‐based phylogenetic tree of Ni‐CODH is mapped with the D‐cluster type information. Figure [file EMI4-18-e70375-s004.docx]

**Supplementary Information**

Genomic survey of carbon monoxide dehydrogenases reveals their widespread distribution in marine habitats

Nipa Chongdar^1,3*^, Anand Goyal^2^, Samir R. Damare^3^

^1^ School of Interdisciplinary Life Sciences, Indian Institute of Technology Goa, ^2^ National Institute of Technology, Warangal, ^3^ CSIR-National Institute of Oceanography

*** Correspondence:** Nipa Chongdar ([nipa@iitgoa.ac.in](mailto:nipa@iitgoa.ac.in))

| **Content** | **Page Number** |
| --- | --- |
| **Table S1.** Ocean zone-wise distribution of Ni-CODHs in different phyla of Archaea | **2** |
| **Table S2:** Ocean zone-wise distribution of Ni-CODHs in different phyla of Bacteria | **2-3** |
| **Table S3.** Ocean zone-wise distribution of MoCu-CODHs in different phyla of Archaea | **3** |
| **Table S4.** Ocean zone-wise distribution of MoCu-CODHs in different phyla of Bacteria | **3-4** |
| **Table S5.** Functional categorization of Ni-CODHs based on their genome context | **4** |
| **Fig S1.** Structures of Ni-and MoCu-CODHs | **5** |
| **Fig S2.** The workflow of the method used to retrieve CODH sequences and curate the dataset | **6** |
| **Fig. S3.** Clade-wise composition of the phyla encoding (A) Ni-CODH and (B) MoCu-CODH | **7** |
| **Fig*.* S4.** Variation of average copy numbers of Mo-CODH genes in Saanich inlet | **8** |
| **Fig*.* S5.** Variation of average copy numbers of Mo-CODH genes in cold-seep water column | **9** |
| **Fig*.* S6.** Variation of average copy numbers of Mo-CODH and Ni-CODH genes in the sediment water interface of *cold seep* site F | **10** |
| **Fig*.* S7.** Variation of average copy numbers of Mo-CODH genes across the San Francisco Coast transect | **11** |
| **Fig S8.** Phylogenetic tree of Ni-CODH showing the bootstrap values | **12** |
| **Fig. S9.** Distribution and co-occurrence of marine Ni-CODH clades across genomes | **13** |
| **Fig S10.** Comparison between CdhA and CooS-type NiCODHs | **14** |
| **Fig*.* S10.** Clade-wise composition of the phyla coding Ni-CODH genes | **14** |
| **Fig*.* S11.** The ML-based phylogenetic tree of Ni-CODH is mapped with the D-cluster type information | **15** |
| **Fig.S12.** Sequence logos and structures of different types of D-clusters observed in our dataset. | **16** |
| **S13.** The ML-based phylogenetic tree of Ni-CODH is mapped with the D-cluster type information in the first layer | **17** |
| **Fig*.* S14.** Distribution of Ni-CODHs from different clades in marine environments | **18** |
| **Fig*.* S15.** The phylogenetic tree of Ni-CODHs with genome context information mapped | **19** |
| **Fig S16.** Schematic representation of the WLP-pathway reaction, enzymes involved, and operon structure | **20** |
| **Fig S17.** Schematic representation of the operon of CODH found in ECH gene cluster | **21** |
| **Fig. S18.** Genomic contexts of Ni-CODH in ACSB-containing, non-WLP genomes. | **22** |
| **Fig S19.** Phylogenetic tree of MoCu-CODH with the bootstrap values | **23** |
| **Fig. S20**. (A) Clade-wise composition of the phyla coding MoCu-CODH genes. (B) Distribution of Mo-CODHs from different clades in marine environments | **24** |

**Table S1:** Ocean zone-wise distribution of Ni-CODHs in different archaeal phyla

| **Phyla** | **Coastal** | **Host Associated** | **Hydrothermal Vent** | **Oilfield** | **Seawater** | **Sediment** |
| --- | --- | --- | --- | --- | --- | --- |
| *Methanobacteriota* | 21 | 0 | 1 | 49 | 1 | 3 |
| *Hadarchaeota* | 0 | 0 | 0 | 1 | 1 | 11 |
| *Halobacteriota* | 51 | 0 | 5 | 87 | 13 | 1 |
| *Asgardarchaeota* | 19 | 0 | 0 | 2 | 3 | 0 |
| *Thermoplasmatota* | 10 | 0 | 0 | 0 | 1 | 0 |
| *Thermoproteota* | 72 | 0 | 0 | 60 | 5 | 5 |
| *Korarchaeota* | 0 | 0 | 0 | 9 | 0 | 0 |
| *Hydrothermarchaeota* | 0 | 0 | 0 | 8 | 0 | 0 |
| *Altiarchaeota* | 3 | 0 | 0 | 2 | 0 | 0 |
| *Nanobdellota* | 2 | 0 | 0 | 3 | 0 | 1 |

**Table S2:** Ocean zone-wise distribution of Ni-CODHs in different bacterial phyla

| **Phyla** | **Coastal** | | **Host Associated** | **Hydrothermal Vent** | **Oilfield** | **Seawater** | **Sediment** | **Solar Saltern** |
| --- | --- | --- | --- | --- | --- | --- | --- | --- |
|  | **Sediment** | **Water** |  |  |  |  |  |  |
| *Acidobacteriota* | 15 | 0 | 0 | 2 | 4 | 1 | 1 | 0 |
| *Actinomycetota* | 7 | 0 | 0 | 4 | 5 | 1 | 2 | 0 |
| *Aerophobota* | 0 | 0 | 0 | 4 | 6 | 0 | 3 | 0 |
| *Aquificota* | 0 | 0 | 0 | 7 | 0 | 0 | 0 | 0 |
| *Armatimonadota* | 3 | 0 | 0 | 1 | 1 | 0 | 0 | 0 |
| *Atribacterota* | 0 | 0 | 0 | 0 | 0 | 0 | 1 | 0 |
| *Bacillota* | 34 | 4 | 5 | 17 | 16 | 10 | 22 | 4 |
| *Bacteroidota* | 0 | 0 | 0 | 1 | 0 | 0 | 1 | 0 |
| *Bipolaricaulota* | 1 | 0 | 0 | 8 | 0 | 0 | 4 | 0 |
| *BMS3Abin14* | 0 | 0 | 0 | 2 | 0 | 0 | 0 | 0 |
| *CAIJMQ01* | 0 | 0 | 0 | 2 | 0 | 0 | 0 | 0 |
| *Campylobacterota* | 0 | 0 | 0 | 0 | 0 | 1 | 0 | 0 |
| *CG2-30-53-67* | 0 | 0 | 0 | 1 | 0 | 0 | 0 | 0 |
| *Chloroflexota* | 80 | 0 | 0 | 52 | 25 | 33 | 33 | 0 |
| *Chrysiogenota* | 0 | 0 | 0 | 2 | 1 | 0 | 0 | 0 |
| *Desulfobacterota* | 347 | 3 | 36 | 282 | 12 | 38 | 56 | 0 |
| *Electryoneota* | 0 | 0 | 0 | 0 | 0 | 0 | 1 | 0 |
| *Hydrogenedentota* | 0 | 0 | 0 | 1 | 0 | 0 | 0 | 0 |
| *Krumholzibacteriota* | 2 | 0 | 0 | 0 | 0 | 0 | 0 | 0 |
| *Latescibacterota* | 0 | 0 | 0 | 2 | 0 | 1 | 0 | 0 |
| *Lernaellota* | 0 | 4 | 0 | 1 | 0 | 0 | 0 | 0 |
| *Myxococcota* | 3 | 0 | 0 | 6 | 0 | 0 | 0 | 0 |
| *Nitrospinota* | 1 | 0 | 0 | 1 | 0 | 0 | 0 | 0 |
| *Nitrospirota* | 10 | 0 | 0 | 25 | 0 | 1 | 2 | 0 |
| *NPL-UPA2* | 0 | 0 | 0 | 0 | 0 | 0 | 1 | 0 |
| *Omnitrophota* | 5 | 0 | 0 | 1 | 0 | 7 | 1 | 0 |
| *Planctomycetota* | 34 | 0 | 0 | 10 | 1 | 18 | 14 | 0 |
| *Poribacteria* | 0 | 0 | 0 | 3 | 0 | 2 | 0 | 0 |
| *Pseudomonadota* | 18 | 1 | 1 | 1 | 0 | 1 | 3 | 0 |
| *Ratteibacteria* | 0 | 0 | 0 | 0 | 0 | 0 | 1 | 0 |
| *SAR324* | 2 | 0 | 1 | 0 | 0 | 4 | 0 | 0 |
| *SM23-31* | 1 | 0 | 0 | 0 | 0 | 0 | 0 | 0 |
| *Spirochaetota* | 8 | 0 | 0 | 4 | 0 | 4 | 2 | 0 |
| *Synergistota* | 0 | 0 | 0 | 1 | 0 | 0 | 0 | 0 |
| *Thermotogota* | 0 | 0 | 0 | 0 | 1 | 0 | 0 | 0 |
| *UBA6262* | 2 | 4 | 0 | 0 | 1 | 0 | 1 | 0 |
| *Verrucomicrobiota* | 2 | 0 | 0 | 3 | 0 | 3 | 1 | 0 |
| *WOR-3* | 0 | 0 | 0 | 1 | 0 | 0 | 2 | 0 |
| *Zhuqueibacterota* | 1 | 0 | 0 | 3 | 0 | 1 | 0 | 0 |

**Table S3.** Ocean zone-wise distribution of MoCu-CODHs in different phyla of Archaea

| **Phylum** | **Coastal** | **Hydrothermal Vent** | **Host Associated** | **Sediment** | **Seawater** | **Solar Saltern** |
| --- | --- | --- | --- | --- | --- | --- |
| *Halobacteriota* | 1 | 3 | 0 | 1 | 2 | 1 |
| *Thermoplasmatota* | 0 | 0 | 0 | 1 | 0 | 1 |

**Table S4.** Ocean zone-wise distribution of MoCu-CODHs in different phyla

| **Phylum** | **Coastal** | | **Hydrothermal Vent** | **Host Associated** | **Sediment** | **Seawater** | **Solar Saltern** |
| --- | --- | --- | --- | --- | --- | --- | --- |
|  | **Sediment** | **Water** |  |  |  |  |  |
| *Pseudomonadota* | 112 | 111 | 7 | 73 | 61 | 68 | 8 |
| *Bacteroidota* | 10 | 7 | 0 | 7 | 5 | 7 | 0 |
| *Actinomycetota* | 33 | 16 | 4 | 89 | 13 | 20 | 2 |
| *Marinisomatota* | 0 | 0 | 2 | 0 | 4 | 5 | 0 |
| *Gemmatimonadota* | 4 | 1 | 0 | 28 | 4 | 1 | 0 |
| *Myxococcota* | 5 | 0 | 1 | 0 | 0 | 1 | 0 |
| *SAR324* | 1 | 4 | 6 | 0 | 10 | 9 | 0 |
| *Bacillota* | 1 | 1 | 1 | 1 | 0 | 0 | 0 |
| *Chloroflexota* | 5 | 0 | 0 | 5 | 5 | 1 | 0 |
| *Deinococcota* | 1 | 0 | 0 | 0 | 0 | 0 | 0 |
| *Desulfobacterota_B* | 0 | 0 | 0 | 0 | 1 | 0 | 0 |
| *Acidobacteriota* | 0 | 0 | 0 | 8 | 2 | 1 | 0 |
| *Verrucomicrobiota* | 0 | 0 | 0 | 2 | 0 | 0 | 0 |
| *Poribacteria* | 0 | 0 | 1 | 0 | 0 | 0 | 0 |

14 bacteria from *Psedomonadota* and 3 from *Actinomycetota* were isolated from unspecified marine sources.

**Table S5.** Functional categorization of Ni-CODHs based on their genome context

| **Functional Category** | **Genome context** (± 15 genes up/downstream) |
| --- | --- |
| WLP | 1. COG1614 is in the genome context. 2. Other *acs*-genes (genes encoding proteins corresponding to COG2069, COG1456, and COG1880 in archaea/COG1410 in bacteria) are present in the genome context, and *COG1614 is in the genome, but at a distant location.* |
| One carbon pool | Have genes formate dehydrogenase (*fdh*, COG3383), or formyl-tetrahydrofolate synthase (*fts*, COG2759), or methylene-tetrahydrofolate dehydrogenase (*folD*, COG0190), or methylene-tetrahydrofolate reductase (*metF­*, COG0685). |
| Incomplete acs cluster | COG1614 is absent in the genome, but any of the other *acs*-subunit genes are present in the gene neighborhood. |
| F_420_-reducing hydrogenase | Have F_420_-reducing hydrogenases (COG3259, COG1035, and COG1941) subunits’ genes in the neighborhood. |
| FNOR | COG1251 |
| Energy-Converting Hydrogenase | COG3260, COG3261, COG0650, COG0651 |
| Hydrogenase maturase | COG0375, COG0378 |
| CooA | COG0664 |
| CooC | COG3640 |
| CooF | COG0437 |
| CooT | COG1532 |
| NADH: ubiquinone oxidoreductase | COG1034, COG1894, COG1905 |
| NitT/TauT family ABC transporter | COG0600, COG0715, COG1116 |
| Heterodisulfide reductase | COG1150, COG1148, COG2048 |
| Glycine-cleavage oxidoreductase | COG0509, COG1249 |

**Fig. S1. Crystal structures of anaerobic and aerobic CODH.** A) The structure of Ni-CODH from Carboxydothermus hydrogenoformans (1suf) found in strictly anaerobic microbes forms a dimer of two identical chains (colored in gray and blue-gray). Each of these chains contains a typical [4Fe4S] cluster (B and B´) and a hybrid FeS cluster with Ni (C and C´). Interfacial FeS cluster (D) is a classical [4Fe4S] cluster ligated by two cysteines from each chain. Inset shows a closer view of the hybrid cluster C that functions as the active center of reversible CO-oxidation. B) Structure of MoCu-CODH from Oligotropha carboxydovorans (1n63). The three subunits of the protein, CoxL (pink), CoxM (blue), and CoxS (green), are shown. The active site and the cofactors responsible for electron transfer (2 x [2Fe-2S] and Flavin Adenine Dinucleotide, FAD) are also shown. A closer view of the bimetallic active center is shown in the inset. The Mo ion is bridged to the Cu center by a sulfido ligand and is also coordinated by a dithiolene ligand from the MCD cofactor and two oxo ligands. The Cu^2+^ ion is bound to the protein matrix via the Cys388 residue. The figures were prepared using PyMOL.

**Fig. S2.** **Workflow for retrieval and curation of Ni-CODH and Mo-CODH datasets.** Protein sequences were retrieved from the NCBI non-redundant database using BLASTp with representative query sequences. Retrieved sequences were filtered based on sequence length, bit score, and redundancy. Final curation included validation of conserved active-site features: Ni-CODH sequences were retained only if all five C-cluster–coordinating cysteine residues were present and if no deletions were observed in regions coordinating the accessory B- and D-clusters. Mo-CODH sequences were validated based on the conserved CoxL active-site motif, allowing for limited sequence variability relative to the canonical motif (AYXCSFR).

**Fig. S3.** Habitat-wise composition of the phyla encoding (A) Ni-CODH and (B) MoCu-CODH genes in the marine ecosystems. For clarity, the phyla with at least 10 representatives are shown in Figure S3A.

**Fig. S4.** Variation of average copy numbers of Mo-CODH genes in Saanich inlet with respect to increasing depth (A), and oxygen (B). Changes in average copy numbers of Ni-CODH genes with depth (C) and oxygen (D)

**Fig. S5.** Variation of average copy numbers of Mo-CODH genes in **cold-seep water column** with respect to increasing depth (A), and oxygen (B). Changes in average copy numbers of Ni-CODH genes with depth (C) and oxygen (D)

**Fig. S6.** Variation of average copy numbers of Mo-CODH and Ni-CODH genes in the sediment water interface of cold seep site F (A) and inside the subsurface sediment (B). Changes in average copy numbers of CdhA and CooS-type Ni-CODH genes with depth in the subsurface sediment (C).

**Fig. S7.** Variation of average copy numbers of Mo-CODH genes across the San Francisco Coast transect with respect to increasing depth (A), and oxygen (B). Changes in average copy numbers of Ni-CODH genes with depth (C) and oxygen (D)

**Fig. S8.** Phylogenetic tree of marine Ni-CODH sequences. The bootstrap values (>0.95) are marked with round labels. Clades A-G are colored according to the scheme shown in the legend. The figure was generated using iTol (Letunic and Bork 2024).

**Fig. S9.** **Distribution and co-occurrence of marine Ni-CODH clades across genomes.**
(A) Bar plots show the number of genomes encoding different counts of Ni-CODH genes (x-axis) across individual phylogenetic clades and in the combined dataset (“Total”), with genome counts on the y-axis; numbers above bars indicate the number of genomes per category. (B) Heatmap showing the conditional probability $P(X\mid Y)$that a genome encodes a Ni-CODH from clade X, given the presence of clade Y. Each row shows, for a given clade Y, how often other clades (X) co-occur within the same genome. Each column shows how often a clade X co-occurs with a given clade Y. Values represent the conditional probability $P(X\mid Y)$.

**Fig. S10**. **Structural comparison of bacterial (CooS) and archaeal (CdhA) Ni-CODHs.** A) Structure of CooS-type Ni-CODH from *Rhodopspirillum rubrum* (9gyb). B) Structure of CdhA-type Ni-CODH from *Methanosarcina thermophila* (9c0r). Like CooS-type enzymes, CdhA contains clusters B, C, and D, and additionally harbors two extra Fe–S clusters (E and F). C) Pairwise structural alignment of the two proteins generated using RCSB and visualized in ESPript (Bittrich et al. 2024; Gouet 2003). Conserved residues are highlighted in red, and residues with similar physicochemical properties are shown in red font.

**Fig. S11.** Clade-wise composition of the phyla coding Ni-CODH genes. The inset shows the same information in percentage format.

**Fig.S12.** **Sequence logos and structures of different types of D-clusters observed in our dataset.** The cysteines involved in coordinating the D-cluster are marked with asterisks in the sequence logo plots. While cysteines coordinating the B-cluster are conserved, the D-cluster shows sequence variation. Representative structures of type I–III D-cluster–containing Ni-CODHs were obtained from the PDB (type I: 9c0r; type II: 9gyb; type III: 6b6v), illustrating the structural plasticity of the D-cluster.

**Fig. S13**. The ML-based phylogenetic tree of Ni-CODH is mapped with the D-cluster type information in the first layer. The second layer represents the isolation information of the Ni-CODHs

**Fig. S14.** **Distribution of Ni-CODHs across phylogenetic clades and marine environments.**
(A) Distribution of Ni-CODHs across clades, showing the number of sequences from different isolation environments within each clade. (B) Distribution of Ni-CODHs across isolation environments, showing the contribution of different clades within each environment.
Insets in both panels show the same data normalized to proportions (fraction of total counts).

**Fig. S15.** The phylogenetic tree of Ni-CODHs with genome context information mapped onto the layer bordering the tree

**Fig. S16.** A) Schemes showing WL-pathway reactions in hydrogenotrophic methanogens (left panel) and acetogens (right). The methanogens and acetogens are typically from the archaeal and bacterial kingdoms, respectively. In the methyl branch, CO_2_ is first reduced to a formate/formyl group and attached to a cofactor, tetrahydromethanoptrein (H4-MPT) in methanogens or tetrahydrofolate (H4F) in acetogens. The formyl group is then further reduced to a CH_3_- group and is transferred onto a corrinoid FeS cluster containing protein (CH3-Co3+/+FeSP). The CH_3_-moiety then reacts with CO, generated by the carbonyl branch, which is similar in both coenzyme A (CoA) to form acetyl-CoA. The corrinoid FeS cluster containing protein is a two-subunit protein in methanogens (CdhDE) and a three-subunit protein in acetogens (AcsDC-AcsE). B) Organisation of typical CODH/ACS genes in archaeal (upper panel) and bacterial (lower panel) genomes. Genes corresponding to cdhB and acsE are unique in archaea and bacteria, respectively.

**Fig. S17**. A) Operon structure of a typical Ni-CODH associated with ECH genes. The genes are marked with COG-IDs and color-coded as follows: genes encoding Ni-CODH and associated proteins are colored green, genes of hydrogenase and associated proteins are colored yellow, and membrane-associated proteins are colored grey. B) The proteins represented by the respective COG-IDs are shown. C) Schematic representation of energy-conserving hydrogenase (ECH) associated Ni-CODH that conserves energy through an ion gradient.

**Fig. S18. Genomic contexts of Ni-CODH in acsB/cdhC-gene containing, non-WLP genomes.** Horizontal bars show the number of genomes assigned to each genomic context based on neighboring gene associations of Ni-CODH. Each genome was assigned a single dominant context when multiple contexts were present, using a predefined priority hierarchy (F_420_-reducing hydrogenase > one carbon pool > energy-converting hydrogenase > FNOR > CooC/CooF/CooT/CooA > glycine cleavage oxidoreductases > heterodisulfide reductase > NADH: ubiquinone oxidoreductase > NitT/TauT family ABC transporter > standalone). Genomes encoding a complete Wood–Ljungdahl pathway (WLP) were excluded from this analysis. **Inset:** Bubble plot showing the distribution of genomic contexts across phylogenetic clades. Each bubble represents a clade–context combination. Bubble size is proportional to the global abundance of the context across all genomes, while grayscale intensity reflects the fraction of genomes within a given clade assigned to that context.

**Fig. S19.** Phylogenetic tree of marine MoCu-CODH sequences. The bootstrap values (>0.90) are marked with round labels. Clades I-VI are colored according to the scheme shown in the legend. This figure was produced using iTol (Letunic and Bork 2024).

**Fig. S20.** (A) Clade-wise composition of the phyla coding MoCu-CODH genes. (B) Distribution of Mo-CODHs from different clades in marine environments.

**Reference**

Bittrich, Sebastian, Joan Segura, Jose M. Duarte, Stephen K. Burley, and Yana Rose. 2024. “RCSB Protein Data Bank: Exploring Protein 3D Similarities via Comprehensive Structural Alignments.” *Bioinformatics* 40 (6): btae370. https://doi.org/10.1093/bioinformatics/btae370.

Gouet, P. 2003. “ESPript/ENDscript: Extracting and Rendering Sequence and 3D Information from Atomic Structures of Proteins.” *Nucleic Acids Research* 31 (13): 3320–23. https://doi.org/10.1093/nar/gkg556.

Letunic, Ivica, and Peer Bork. 2024. “Interactive Tree of Life (iTOL) v6: Recent Updates to the Phylogenetic Tree Display and Annotation Tool.” *Nucleic Acids Research* 52 (W1): W78–82. https://doi.org/10.1093/nar/gkae268.
